# The effect of an eye mask on midazolam requirement for sedation during spinal anesthesia: a randomized controlled trial

**DOI:** 10.1186/s12871-021-01451-1

**Published:** 2021-09-25

**Authors:** Seon Woo Yoo, Min-Jong Ki, Dal Kim, Yu Jin Oh, Jeongwoo Lee

**Affiliations:** 1 Department of Anesthesiology and Pain Medicine, Jeonbuk National University Medical School and Hospital, 20, Geonji-ro, Deokjin-gu, Jeonju, 54907 South Korea; 2 Research Institute of Clinical Medicine of Jeonbuk National University-Biomedical Research Institute of Jeonbuk National University Hospital, Jeonju, South Korea

**Keywords:** Bispectral index, Midazolam, Modified Observer Assessment of Alertness and Sedation Scale, Sedation, Spinal anesthesia

## Abstract

**Background:**

Midazolam is frequently used for sedation during spinal anesthesia. However, external environmental factors, such as bright surgical lights, may hamper patient relaxation, which may lead to an increase in the dose of midazolam required and the likelihood of adverse drug effects. We investigated whether using an eye mask to block the external environment could reduce midazolam requirements during spinal anesthesia.

**Methods:**

Participants aged 18–‒80 years, scheduled for elective surgery under spinal anesthesia, were randomly divided into a masked group (wearing eye masks during surgery, *n* = 20) and a control group (no mask, *n* = 18). The sedation level was assessed using a modified Observer Assessment of Alertness and Sedation (MOAA/S) scale. Midazolam (1 mg) was incrementally administered every 5 min until moderate sedation (MOAA/S score of 3) was achieved. The bispectral index (BIS) was monitored, and the onset and maintenance times of a BIS < 80 were recorded.

**Results:**

The two groups had similar demographic characteristics. The midazolam requirements were significantly lower in the masked group than in the control group (2.8 mg vs. 3.7 mg, *P* = 0.024). However, the onset and maintenance times for a BIS < 80 were similar. In addition, there were no significant differences in the incidence of side effects or patient satisfaction between the two groups.

**Conclusions:**

Blocking the external environment with an eye mask during spinal anesthesia can reduce the requirement for sedatives, such as midazolam.

**Trial registration:**

The trial was retrospectively registered with the Clinical Research Information Service (No. KCT0005528, 15/10/2020) entitled “Can we reduce an amount of sleeping pills just by blocking light?”.

## Background

During regional anesthesia, intravenous sedatives are commonly administered to patients for various reasons. The sedatives relieve the patient’s anxiety and induce hypnosis, making the patient more comfortable during surgery [1]. However, various environmental factors in the operating room may interfere with patient relaxation. Noise made by medical staff and surgical instruments in the operating room, bright lights, and unfamiliar environments increase patients’ anxiety. For this reason, it is often difficult to reach a state of sufficient sedation, despite the administration of sedatives. This may impose a need for higher doses of sedatives, which could increase the likelihood of associated adverse drug effects.

Among various sedatives, midazolam has long been used during spinal anesthesia because of its advantages of a faster onset of action, short duration of hypnosis, and ease of use [2, 3]. Midazolam, similar to other benzodiazepines, causes dose-dependent respiratory depression by reducing muscle tone and the breathing stimulatory effect of carbon dioxide [4, 5]. Even a small dose of anxiolytics can depress the hypoxic ventilation response in humans [6]. In particular, spinal anesthetics are known to decrease the requirement for midazolam [7], and their combined administration with opioids and other sedatives results in a modest synergistic effect in the depression of resting ventilation [8, 9]. In clinical practice, unexpected overdoses of midazolam during spinal anesthesia require the attention of medical staff in the operating room, recovery room, or even the ward [10]. Consequently, many anesthesiologists are reluctant to administer sufficient amounts of midazolam during spinal anesthesia.

We hypothesized that if conditions that interfere with patient sedation in the operating room could be blocked, the appropriate level of sedation could be achieved more easily and safely, without excessive administration of hypnotics. There have been many studies on the dose and side effects, as well as comparisons and combinations of sedatives, but few studies have investigated how to reduce the dose of sedatives and still achieve the desired level of sedation. Therefore, we aimed to investigate the auxiliary sedation effect of using an eye mask to block strong light in patients undergoing surgery. Specifically, we sought to determine whether and to what extent the use of an eye mask by the patient could reduce the amount of midazolam required to achieve sufficient sedation in patients undergoing surgery during spinal anesthesia.

## Methods

### Patients and ethics

This prospective, randomized, controlled trial was approved by the relevant hospital ethics committee (Jeonbuk National University Hospital Institutional Review Board, registration no. CUH 2017–06-037) and retrospectively registered with the Clinical Research Information Service (CRIS) (No. KCT0005528, 15/10/2020). The study included 40 adult patients aged 18–80 years who were scheduled for elective surgery under spinal anesthesia at a tertiary care hospital between August 2017 and August 2018. The exclusion criteria were as follows: American Society of Anesthesiologists physical status (ASA PS) ≥ 3, pregnancy, sleep disorders such as sleep apnea, intellectual disability, or any contraindication to spinal anesthesia. Patients who refused to take sedatives or wear an eye mask were also excluded. In the afternoon on the day before surgery, one of the researchers visited the ward of the study subject, judged their eligibility, and went through an explanation and consent procedure. All patients read the consent form by themselves and were able to consent in writing based on their voluntary judgment. After obtaining informed consent, 40 patients were enrolled and randomly assigned to two parallel groups of 20 patients each in a 1:1 ratio. The group wearing an eye mask was defined as the masked group, and the group without an eye mask was used as the control group. The study was performed in accordance with the Ethical Principles for Medical Research Involving Human Subjects, outlined in the Declaration of Helsinki of the World Medical Association.

### Study protocol

Routine premedication was not administered. All patients were evaluated with an electrocardiogram, noninvasive blood pressure monitoring system, and pulse oximeter, and a 20-gauge intravenous catheter was inserted into the patient’s forearm. We injected 10‒14 mg of 0.5% hyperbaric bupivacaine (Marcaine-Heavy® 5 mg/mL, AstraZeneca, Seoul, Korea) intrathecally via a 25-gauge spinal needle for spinal anesthesia. All techniques were performed using a midline or para-median approach in the lateral decubitus position. The patient was placed in the supine position, and nasal prong with capnography and bispectral index (BIS) monitoring devices were applied. The sensory block level was evaluated using the cold skin test, and the Trendelenburg position or the reverse Trendelenburg position was used as appropriate to control the thoracic sensory block level T6–10. Twenty minutes after bupivacaine administration, the block height was re-evaluated using the cold skin test and recorded. An eye mask was then applied to the masked group.

Firstly, to measure the dose of midazolam required for sedation, the main outcome of the study, intravenous midazolam (1 mg) was incrementally administered every 5 min, and the level of sedation was repeatedly evaluated using a modified Observer Assessment of Alertness and Sedation (MOAA/S) scale (scored as follows: 5 = responds readily to name spoken in normal tone; 4 = lethargic response to name spoken in normal tone; 3 = responds only after the name is called loudly or repeatedly; 2 = responds only after mild prodding or shaking; 1 = responds only after painful trapezius squeeze, 0 = no response after painful trapezius squeeze). We used only scores of five, four, and three out of the six possible MOAA/S scale categories. One researcher spoke the patient's name in the same normal tone to evaluate the MOAA/S score every 5 min after midazolam administration. If the patient responded readily and normally, a MOAA/S score of 5 was assigned; if the response was lethargic, a MOAA/S score of 4 was assigned, and if the patient did not respond, a MOAA/S score of 3 was given. There was no additional stimulation such as loud or repetitive naming, mild prodding or shaking, or painful squeezing; therefore, MOAA/S scores < 3 were not identified. We defined a MOAA/S score of 3 as “sufficient sedation” and MOAA/S scores of 4 or 5 as “insufficient sedation,” and if the patient was in an “insufficient sedation” state, an additional 1 mg of midazolam was administered until “sufficient sedation” was achieved. This was repeated every 5 min for a maximum of 35 min, and the time to reach an MOAA/S score of 3 and the dose of administered midazolam were recorded. If “sufficient sedation” was not reached by 35 min (7 mg of midazolam), the outcome was defined as “sedation failure,” and the time was recorded as 40 min. For consistency, all evaluations were performed by a single researcher (Dr. S. W. Yoo).

We also evaluated the BIS at the same time points as the MOAA/S scores were assessed. Firstly, to evaluate the onset of sedation more objectively, the time to reach a BIS < 80 was measured. Secondly, the duration of sedation was evaluated by measuring the length of time from the onset of a BIS < 80 until the BIS returned to a level > 80. Maintenance time was excluded if the operation was completed before spontaneous awakening of the patient. In addition, vital signs were recorded from the time of midazolam administration until the end of surgery. Twenty minutes after bupivacaine administration (at 0 min of midazolam administration) was defined as baseline. Systolic blood pressure and heart rate decreases by > 20% from this baseline were regarded as the development of hypotension and bradycardia, respectively. Transient hypotension induced by tourniquet release was also excluded. Apnea was defined as the repeated interruption of airflow for > 10 s based on capnography. After surgery, all patients were transferred to the recovery room, and approximately 30 min later, one of the researchers assessed patient satisfaction with sedation (3 = very good, 2 = good, 1 = not bad, 0 = bad).

### Statistical analysis

When the difference in midazolam dose between the two groups was estimated using G Power 3.1.9.7 software’s independent *t*-test sample size calculation, with an average of 1 mg and a standard deviation of 1 mg based on a previous study [2], we determined that 36 patients were required to achieve a significance level of 0.05, and a power of 90%. Considering a 10% dropout rate, we included 40 patients. Block randomization was used to minimize imbalance in the number of patients between the groups. On the morning of the surgery, the researcher scheduled to perform the experiment assigned the patient to the masked or control group by picking one stone from a container containing 20 white stones and 20 black stones. Patients with black stones were assigned to the masked group and those with white stones were assigned to the control group. Statistical analyses were performed using IBM Statistical Package for the Social Sciences (SPSS) Statistics v. 26 software (SPSS, Chicago, IL, USA). Data are expressed as the mean ± standard deviation or number of patients. The primary outcome was the difference between the two groups in the dose of midazolam required to achieve an MOAA/S score of 3 during spinal anesthesia, and the secondary outcome was a comparison between the two groups for the onset time to reach a BIS < 80 and the duration of time the BIS was < 80. The independent *t*-test or Mann–Whitney U test was used depending on whether or not the data were normally distributed for continuous variables, and the χ^2^ test or Fisher’s exact test was used for categorical variables. The cut-off for statistical significance was set at *P* < 0.05.

## Results

**Fig. 1:**
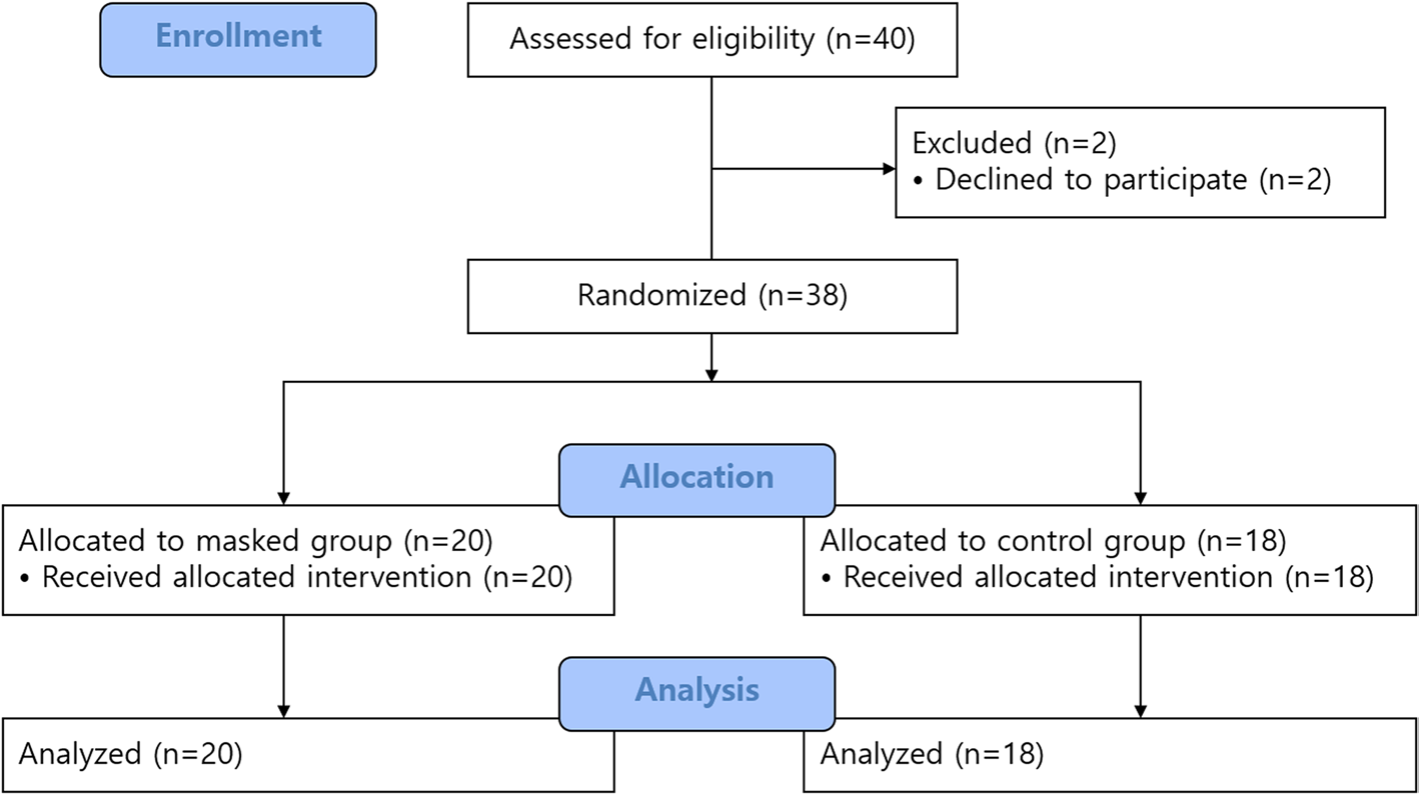
CONSORT flow chart of patients

Of the 40 patients enrolled, two were excluded for refusing spinal anesthesia on the day of surgery. Consequently, 38 patients were included in the analysis (Fig. 1). There were no statistical differences between the masked and control groups in terms of baseline characteristics (Table 1).

**Table 1:** Baseline patient characteristics

	**Masked group** **(*n* = 20)**	**Control group** **(*n* = 18)**	***P*-value**
Age (years)	56.8 ± 14.5	52.2 ± 18.1	0.392
Sex (male/female)	11/9	9/9	0.758
Height (cm)	160.4 ± 8.2	161.8 ± 11.0	0.650
Weight (kg)	65.8 ± 8.9	72.9 ± 16.7	0.106
ASA PS classification (I/II)	10/10	7/11	0.492
Bupivacaine dose (mg)	12.6 ± 1.2	12.9 ± 1.3	0.331
Sensory block level [n (%)]			0.907
Below T7	3 (15.0)	4 (22.2)	
T7‒T9	5 (25.0)	5 (27.8)	
Above T9	12 (60.0)	9 (50.0)	
Baseline BIS	94.2 ± 3.5	93.9 ± 4.8	0.806
Operation time, total (min)	71.1 ± 37.7	65.3 ± 47.0	0.443
Type of surgery			
Knee arthroplasty [n (%)]	7 (35.0)	4 (22.2)	0.386
Other orthopedic surgery [n (%)]	11 (55.0)	12 (66.7)	0.463
Urological surgery [n (%)]	2 (10)	2 (11.1)	0.656

Values are presented as the mean ± standard deviation

*ASA PS* American Society of Anesthesiologists Physical Status, *BIS* bispectral index

**Fig. 2:**
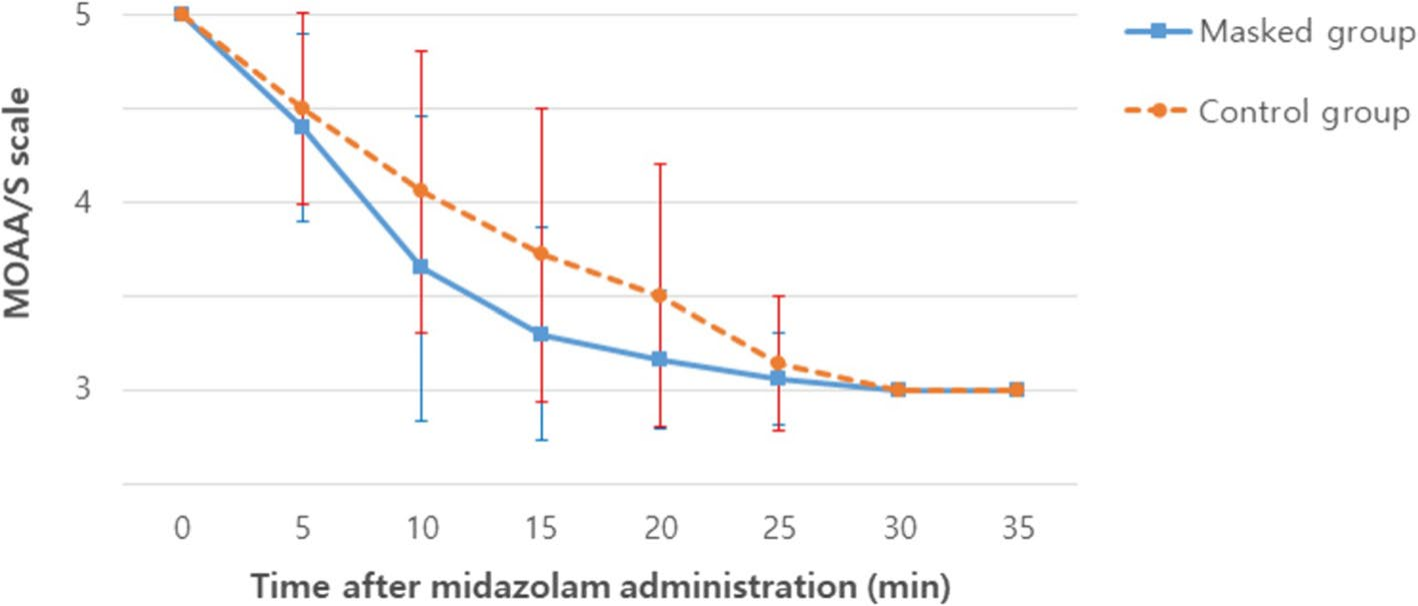
Evaluation of the MOAA/S score over time, from the initial midazolam administration to 35 min later. Data represent the mean ± standard deviation (SD). MOAA/S, modified Observer Assessment of Alertness and Sedation. *P < 0.05, masked group vs. control group

In the masked group and the control group, the midazolam doses required to reach an MOAA/S score of 3 were 2.8 mg and 3.7 mg, respectively. The masked group required a significantly lower midazolam dose than the control group (*P* = 0.024) (Table 2). Likewise, the time to reach this score was also significantly shorter in the masked group than in the control group (*P* = 0.024). Figure 2 shows the change in the MOAA/S score at each time point in the two groups. The onset time to reach a BIS < 80 corresponded with doses of 12.3 mg and 14.7 mg in the masked group and the control group, respectively; these values did not significantly differ between the two groups (*P* = 0.264). Similarly, the duration of time in which the BIS was maintained at < 80 corresponded to 28.6 min in the masked group and 21.4 min in the control group, with no significant differences between groups (*P* = 0.183).

**Table 2:** Comparison of data between the two groups after midazolam injection

	**Masked group** **(*n* = 20)**	**Control group** **(*n* = 18)**	***P*-value**
Total dose of midazolam administered (mg)	2.8 ± 1.0	3.7 ± 1.3	0.024
Onset time to reach an MOAA/S score = 3 (min)^a^	13.8 ± 4.8	18.3 ± 6.4	0.024
Onset time to reach a BIS < 80 (min)^b^	12.3 ± 5.5	14.7 ± 6.3	0.264
Time maintaining a BIS < 80 (min)	28.6 ± 16.7	21.4 ± 21.0	0.183
Sedation failure [n (%)]^c^	0 (0)	0 (0)	1.000
Incidence of relative hypotension [n (%)]^d^	1 (5)	3 (16.7)	0.328
Incidence of relative bradycardia [n (%)]^e^	0 (0)	1 (2.6)	0.474
Incidence of apnea [n (%)]^f^	2 (10.0)	4 (22.2)	0.395
Score of patient satisfaction with sedation ^g^	2.6 ± 0.8	2.6 ± 0.6	0.633

Values are presented as means ± standard deviations

*Abbreviations*: *MOAA/S score* modified Observer Assessment of Alertness and Sedation score, *BIS* bispectral index

^a^ Time to achieve an MOAA/S score of 3 following midazolam administration

^b^ Time to reach a BIS < 80 following midazolam administration

^c^ An MOAA/S score of 3 was not reached when midazolam was administered every 5 min for 35 min

^d^ Systolic blood pressure decreased by > 20% from baseline (0 min of midazolam administration)

^e^ Heart rate decrease by > 20% from baseline (0 min of midazolam administration)

^f^ Repeated interruption of airflow for at least 10 s based on capnography (respiration rate < 6 breaths/min)

^g^ Very good = 3, good = 2, not bad = 1, bad = 0

There was no significant difference in the incidence of relative hypotension or bradycardia between the masked group and the control group (*P* = 0.328 and *P* = 0.474, respectively), nor was there any significant difference in the incidence of apnea between groups (*P* = 0.395). Furthermore, there was no significant difference in patient satisfaction with sedation between the masked and control groups (*P* = 0.588). None of the patients experienced sedation failure.

## Discussion

Previous studies have focused on the utility of midazolam, its side effects, and its comparison with other drugs such as dexmedetomidine, propofol, and opioids. However, to date, no study has evaluated the effect of external environmental factors on patient sedation, which is the main purpose of sedative drugs. This is the first study to compare the requirement of sedatives while controlling external environmental factors. In this study, we investigated whether blocking some external environmental factors (bright surgical lights) could reduce the amount of midazolam required for adequate sedation in patients undergoing surgery under spinal anesthesia. The results of this study show that the masked group required a significantly lower dose of midazolam to reach an MOAA/S score of 3 compared to the control group. When 1 mg of midazolam was administered every 5 min, the doses of midazolam required to reach a MOAA/S score of 3 were 2.8 mg and 3.7 mg in the masked group and the control group, respectively, a difference of about 0.9 mg. We found that the midazolam dose could be reduced by controlling the amount of bright light in the environment. Although there were no significant differences between groups based on BIS measurements, a faster onset and a longer duration of sedation were observed in the masked group than in the control group. This suggests the need for further studies with larger sample sizes.

Although midazolam is the most frequently used sedative for minor surgeries or procedures, it is difficult to titrate the ideal dose because of large individual variability in responses [11]. According to certain recommendations, midazolam is usually administered intravenously at incrementally increasing doses of 0.5 mg or 1 mg until the desired degree of sedation is achieved [12]. This incremental approach is used to avoid serious side effects, such as hypoventilation, respiratory depression, cardiovascular suppression, and delirium, which accompany midazolam overdose [4, 5]. Moreover, the elderly, obese, and patients with hepatic or renal disease are at higher risk of these side effects [13–15], and midazolam also has synergistic effects with other drugs, such as hypnotics or opioids [16, 17]. Our study results could provide a good guide for safety improvements in future midazolam use.

In terms of the onset time to reach a BIS ≤ 80, the masked group and the control group required 12.3 min and 14.7 min, respectively, which was faster than the times required to reach an MOAA/S score of 3 of 13.8 min and 18.3 min, respectively. This is probably because a BIS of 80 does not reflect an MOAA/S score of 3. According to the manufacturer’s report, a BIS of 80 corresponds to “Light/moderate sedation, may respond to loud commands or mild prodding/shaking,” which is similar to the MOAA/S score of 3 [18]. However, when we compared each MOAA/S score with the BIS values in this study, a BIS of 80 was closer to a MOAA/S score of 4. The average BIS values of those with MOAA/S scores of 5, 4, and 3 were 89.3, 80.3, and 70.0, respectively. To date, no large-scale study has directly compared the relationship between BIS values and MOAA/S scores.

Intrathecal local anesthetic appears to stop spreading 15‒25 min after injection [12, 19, 20]. Thereafter, changes in blood pressure, pulse rate, and respiratory rate are thought to be mainly related to the effects of midazolam if other variables are controlled [21]. The incidence of these side effects was higher in the control group than in the masked group, but the difference was not significant. Since the number of patients with these side effects in our study was small, larger studies are needed. There was no significant difference in patient satisfaction with sedation between the two groups. The reasons for low satisfaction were frequent blood pressure measurements, fixed arms, noise, bright lights, etc. In rare cases, some patients felt uncomfortable with the eye mask and had a fear of covering their eyes.

This study had several limitations. Above all, our study was not a blinded trial, as the researcher was inevitably able to distinguish whether the patient was wearing an eye mask or not. Therefore, it was not possible to completely prevent the risk of bias, especially detection bias. In addition, the type of surgery differed across patients, and variables such as noise were not controlled for. We only classified the MOAA/S scores of 5, 4, and 3, but not those < 3, for the following reasons: first, we did not want to harm the patient with unnecessarily deep sedation beyond the light/moderate sedation required. Second, other stimuli, such as speaking loudly, prodding, or shaking, to classify MOAA/S scores < 3, may have affected the results by interfering with the patient’s sedation. Third, there was a limitation in evaluating the response of the patient’s eye opening in the masked group, and thus the judgment of the MOAA/S score could have differed between the two groups.

## Conclusions

An eye mask is an easily used, noninvasive, and cost-free method for reducing external anxiety-causing stimuli. Our findings suggest that blocking light using an eye mask during spinal anesthesia can reduce the requirement for sedatives such as midazolam. In the future, additional studies will be necessary to verify the reproducibility of the present findings and to examine the relationships between various drugs and environmental factors.

## Data Availability

The datasets used and/or analyzed during the current study are available from the corresponding author upon reasonable request.

## Abbreviations

ASA PS: American Society of Anesthesiologists physical status
BIS: Bispectral index
CRIS: Clinical Research Information Service
MOAA/S: Modified Observer Assessment of Alertness and Sedation
